# Predicting Offenders' Institutional Misconduct and Recidivism: The Utility of Behavioral Ratings by Prison Officers

**DOI:** 10.3389/fpsyt.2018.00679

**Published:** 2018-12-10

**Authors:** Joscha Hausam, Robert J. B. Lehmann, Klaus-Peter Dahle

**Affiliations:** ^1^Institute of Forensic Psychiatry, Charité – Universitätsmedizin Berlin, Berlin, Germany; ^2^Department of Psychology, Medical School Berlin, Berlin, Germany; ^3^Department of Psychology, University of Hildesheim, Hildesheim, Germany

**Keywords:** behavior rating scale, SWAP-200, prison behavior, behavioral observation, risk assessment, correctional treatment, prison officers

## Abstract

Measures of current behavior are rarely incorporated into risk assessment. Therefore, the current study used a behavior rating scale to assess prison officers' observations of inmates prison behavior and examined the contribution of these ratings for risk assessment. Prison officers rated 272 sexual and violent offenders in three different correctional treatment facilities in Berlin, Germany. Factor analysis revealed three psychologically meaningful factors measuring externalizing, internalizing and adaptive prison behavior. The construct validity of the three factors was established through correlational analyses with standardized risk assessment instruments. Externalizing and internalizing behaviors were significant predictors of violent recidivism after release. In addition, externalizing was a significant predictor of institutional misconduct, whereas adaptive and internalizing behavior predicted whether an inmate was granted privileges (e.g., minimum-security confinement). Logistic regression analyses indicated that externalizing behavior ratings added incrementally to the Level of Service Inventory-Revised for the prediction of institutional misconduct and violent recidivism. The results indicate that prison officers observe important prison behaviors and that behavioral ratings can improve risk assessment.

## Introduction

Forensic risk assessment requires collecting diverse information. Although the value of behavioral assessment has been recognized (1, 2), only few attempts have been made to systematically incorporate measures of current behavior into risk assessment. This paper investigates the validity of a behavior rating scale assessed by prison officers. The greater goal of this research question is to use these ratings to improve risk assessment in correctional treatment services.

Informed risk assessment should focus on individual risk factors that are theoretically and empirically linked to recidivism [e.g., (3)]. Risk factors have often been classified as either static (i.e., generally unchangeable) or dynamic (i.e., amenable to change). Mann et al. (4) proposed to adopt the concept of psychologically meaningful risk factors instead. Both static (e.g., criminal history) and dynamic risk factors (e.g., criminal attitudes) predict recidivism, because they are markers for the same underlying individual propensities (e.g., antisocial orientation). Propensities are considered—like personality traits—to be relatively enduring offender characteristics that “may or may not manifest during any particular time period” [(4) p. 194]. Behavioral consistency is more likely to occur across situations when similar psychological characteristics are triggered (5).

Jones (2004)(6) recently introduced the framework of offense paralleling behavior (OPB) to identify risk-related current behavior. The central assumption is to identify behavioral patterns or sequences that share functional similarity to prior offense behavior. It has been suggested that propensities may reveal themselves through observations of offense paralleling behavior 7. Using a qualitative approach, Atkinson and Mann (8) found strong congruence between prison officers' observations (e.g., resistance to rules and supervision) and empirically established risk factors (e.g., antiauthority). The authors conclude that “these types of observations could, if utilized appropriately, improve the process of forensic psychological risk assessment; specifically in relation to focusing on current functioning to complement traditional forensic methods which tend to focus on past behavior” [(8), p. 152]. Consequently, it should be possible to identify risk-related behavior in prison with a rating scale assessed by prison officers.

Behavior rating scales are one of the most frequently used assessment measures in psychological research and practice. They provide a quick and reliable account of specific behaviors for diagnostic and intervention planning purposes. Behavior rating scales are considered objective measures with many advantages when administered to an informant who is familiar with the subject [see Merrell (9)]. For the purpose of the present study, we outline two specific advantages of behavior ratings scales for the use with incarcerated offenders. First, behavior rating scales can be used to address behavioral or personality characteristics of offenders who cannot (e.g., lack of insight) or do not want (e.g., impression management or malingering) to provide valid information about themselves. In this context, external ratings are not susceptible to “self-serving cognitive distortions” (10), which are considered as risk factors themselves for general (11) and sexual recidivism (4). For example, Milton et al. (12) compared staff and self-report ratings of interpersonal functioning and reported that, compared to staff ratings, offenders tended to underestimate their dominance and coerciveness, and overestimated their nurturance. Second, rating scales offer standardized means to what degree a specific behavior is present and allow for a “statistical aggregation of standardized clinical observations” [(13); p. 598]. Unlike checklists, behavior ratings scales assess the frequency of observed behavior on a Likert-type scale (e.g., never, sometimes, always). Therefore, they provide quantifiable and normative data, which can be used to compare ratings of different groups or across settings (14). They can also be used to track individual behavioral changes over the course of time, e.g., following treatment. Concerning offender treatment, observable changes of risk-relevant behaviors may serve as an indicator for reductions in reoffending.

Prison officers have the greatest amount of daily interaction with inmates and therefore know them quite well. They are more readily available than therapeutic staff and constitute important agents in crisis intervention and treatment delivery (15). Furthermore, Atkinson and Mann (8) proposed that prison officers are experienced behavioral observers and are a valuable but untapped source for risk assessment purposes. Few attempts have been made to examine observer ratings in offender populations. Quay (16) developed the Adult Internal Management System (AIMS) for internal classification to effectively deal with different types of prisoners. The system attempts to identify five different types of prisoners based on historical information and behavioral ratings by correctional officers: the aggressive-psychopathic, the manipulative, the normal (situational), the inadequate-dependent, and the neurotic-anxious prisoner. However, studies only found three distinct groups, the aggressive-manipulative, the normal, and the weak prisoner (17, 18). Subsequently, Cooke (19) developed the Prison Behavior Rating Scale (PBRS) to assess psychological features of disturbed behavior in prison. The PBRS consists of 36 items and 3 subscales: Antiauthority (e.g., aggressive toward staff), Anxious-Depressed (e.g., frightened of other inmates), and Dull-Confused (e.g., appeared sluggish and drowsy). While the evidence for the latter two scales was less compelling, the Antiauthority scale showed utility in the prediction of institutional misconduct (20).

The Chart of Interpersonal Reactions in Closed Living Environments [CIRCLE; (1)] is a staff rating scale (e.g., nurses in forensic hospitals) developed to assess an individual's social behavior according to the interpersonal circumplex (IPC). Briefly summarized, the IPC assumes that two orthogonal dimensions, status (dominance vs. submission) and affiliation (hostility vs. nurturance), define interpersonal behavior (21). The CIRCLE assesses eight interpersonal styles and is the most widely used behavior rating scale in offender samples. It is reported to have satisfactory psychometric and circumplex properties (22). Previous research with offenders has highlighted the theoretical and empirical importance of the interpersonal patterns denoted as dominant, coercive, and hostile. Specifically, these CIRCLE scales were predictive of institutional misconduct and violence in mentally disordered offenders in forensic hospitals (23–25) and prison (26). It was also suggested that the dominant, coercive, and hostile scales of the CIRCLE are linked to cluster B personality disorders, such as antisocial, histrionic, and narcissistic (27).

Only recently, Hausam et al. (28) reported preliminary results on behavioral ratings by prison officers in a small juvenile sample (*N* = 62). The scales were developed based on theoretical considerations and showed acceptable values of internal consistency and inter-rater reliability. Correlational analyses using different indexes (e.g., age and violent behavior in prison) and risk assessment instruments (e.g., HCR-20) attested to the construct validity of the scales. Furthermore, correctional officers' ratings were predictive of treatment attrition. For a smaller subsample, ratings at two time points (after 1 year) were available. Results indicated that prison officers are generally able to track positive and negative behavioral changes during treatment. The current study extends these findings taking the extensive research of the Shedler-Westen Assessment Procedure [SWAP-200; (29)] into account. The SWAP-200 allows for a comprehensive assessment of personality and personality pathology in psychiatric (30) and forensic populations (31). Recent studies have shown that the SWAP-200 assessment is associated with institutional (mis-) behavior (as measured with the CIRCLE) in psychiatric patients (32) and personality-disordered offenders (27). The SWAP-200 was modestly predictive of inpatient violence (31).

We propose that prison officers with special training for correctional treatment are experienced observers and are likely to be a valuable supplement for forensic assessment. In Germany, correctional treatment units mostly follow a therapeutic community-based approach of rehabilitation. The prison officers are part of the therapeutic community to surveil, supervise, and support inmates on a daily basis. Consequently, prison officers‘ experiences and knowledge of inmates' behavior is often embedded in regular case management routines (e.g., parole release decisions). However, the units often use unsystematic behavioral checklists or rely on experience reports, which must be considered critical for two reasons. First, prison officers do observe risk-relevant behavior that may not be reported (8). Second, clinical observations are more beneficial if used systematically (13).

### Purpose of Study

The aim of the present study was to investigate the applicability and validity of the SWAP rating scale (SWAP-RS) in three different correctional treatment samples. First, factor structure of the SWAP-RS will be examined. This is considered the most important step to establish construct validity (33). We hypothesized to find a factor structure similar to the factors of the SWAP-200 (34). Second, the construct validity of the factors thus identified will be tested by examining associations with standardized risk assessment instruments. Third, the predictive validity of behavioral ratings by prison officers will be investigated. Fourth, the incremental validity of the ratings in predicting institutional (mis-) conduct and recidivism beyond risk assessment instruments will be tested.

## Methods

### Sample

The sample was composed of *N* = 272 male offenders of three different correctional treatment units in Berlin, Germany. Specifically, the subsamples were collected from social-therapeutic units for adults (*n* = 145) and juveniles (*n* = 75), as well as a preventive detention unit (*n* = 52). These units generally follow a group-based approach of rehabilitation and encompass a mix of individual and group therapy, social skills training, and educational or vocational training. Apart from therapeutic staff, specifically trained prison officers are part of these units to surveil, supervise, and support prisoners. Therefore, they largely define the field of social experience, know their inmates quite well, and are experienced observers of offender behavior in prison. At the point of rating, the inmates were 37.52 years old (*SD* = 14.70; Range = 16.91–81.97) and incarcerated for 59.36 months (*SD* = 59.74; Range = 1.64–364.32). *N* = 30 inmates (11%) were convicted of murder or manslaughter, *n* = 99 (36.4%) of robbery or assault, *n* = 60 (22.1%) of rape, *n* = 71 (26.1%) of sexual abuse, and *n* = 12 (4.5%) of other offenses. The inmates had an average sentence length^1^ of 6.18 years (*SD* = 4.56; Range = 1.5–25) and on average six prior convictions (*SD* = 5.62; Range = 0–34).

### Procedure

Data was collected between 2014 and 2016 as part of an on-going evaluation project. The evaluation project was carried out in accordance with the recommendations of the Senate for Justice, Consumer Protection and Anti-Discrimination of Berlin, Germany. Ethical approval for the study was sought and granted by the Ethics Committee of Charité—Universitätsmedizin Berlin (EA4/131/18). All participants gave written informed consent in accordance with the Declaration of Helsinki. The protocol was approved by the Official Data Protection Officer of Charité—Universitätsmedizin Berlin.

Prison officers were asked to rate all inmates admitted to one of the three units during that time (response rate: 80.1%). Group meetings with the prison officers at several time points during data collection were arranged to communicate general information about the study (e.g., that inmates should be rated by prison officers who are familiar with them, anonymization procedure, etc.). The officers did not receive special training in the assessment of the rating scale. A total of 76 prison officers rated on average three inmates (*M* = 3.32, *SD* = 2.37, Range = 1–12) they have known for *M* = 18.76 months (*SD* = 23.03, Range = 1–156).

### Measures

#### SWAP Rating Scale

Inmate behavior was assessed using the SWAP rating scale (SWAP-RS). The SWAP-RS is a shortened adaptation of the items of the Shedler-Westen Assessment Procedure-200 [SWAP-200; (29); German version: (35)]. The SWAP-200 is a valid tool for personality assessment and consists of 200 personality-descriptive statements. It is a clinician-rated instrument with items that are suitable for external rating. The items are written in clear and jargon free language designed to assess, quantify, and compare clinical observations (29). The procedure allows for a categorical diagnosis based on the Q-sort method and a dimensional measurement of 12 factors based on a numeric value [see (34)]. A 5-point Likert-type scale was chosen to assess frequency of observed behavior (never, rarely, occasionally, frequently, and very frequently observed; scored 0 to 4). Prison officers were instructed to rate an inmates' behavior according to their observations. As mentioned before, we sought to assess risk-related propensities that manifest in current behavior. Based on empirical [i.e., factor loadings; (34)] and theoretical considerations (i.e., appropriateness for prison context), we included five items each of the following factors: Psychopathy^2^, hostility, narcissism, emotional dysregulation, dysphoria, and schizoid orientation. In addition, 10 items of the psychological health factor were included as well. The factors psychopathy (e.g., reckless and unlawful behavior), hostility (e.g., chronic anger and mistrust), narcissism (e.g, self-importance and arrogance), and emotional dysregulation (e.g., emotions tend to change rapidly and unpredictably) seem to be associated with the risk-related propensity of antisocial orientation [e.g., (11, 37)]. The factors dysphoria (e.g., feeling inadequate, avoids social situations) and schizoid orientation (e.g., lacks close relations and social skills) are composed of internalizing characteristics. Some of these features were identified being risk-relevant for general [e.g., (38)] and sexual recidivism [e.g., (4)]. Finally, the factor psychological health includes strengths and resources, or stated differently, they may refer to positive behaviors in prison (6). They may be considered as protective factors. A growing body of research emphasizes the complementary use of risk and protective factors in risk assessment (39).

#### Risk Assessment

Professionally trained psychologists independent of the treatment units completed ratings on the Level of Service Inventory—Revised [LSI-R; (38); German version: (40)] the Historical-Clinical-Risk Scheme [HCR-20; (41); German version: (42)], and the Psychopathy Checklist—Revised [PCL-R; (36)] based on file review. The LSI-R was selected as a measure of general risk of recidivism, the HCR-20 as a measure of risk of violent recidivism, and the PCL-R as measure of the psychopathy construct, which has shown to be a robust predictor of persistent delinquency. Predictive validity of the measures is well documented, also in German speaking samples [e.g., (43)].

#### Institutional Behavior

A follow-up review of inmate files was conducted after *M* = 17.69 months (*SD* = 10.71, Range = 3.65–57.53) by the members of the research group to collect data on different outcome measures of institutional behavior. These included the absence/ presence of violent (e.g., physical aggression) and non-violent disciplinary misconduct (e.g., possession of prohibited items). In addition, we assessed whether an inmate was granted privileges, such as temporary release, outside employment, or minimum-security confinement. Frequencies were 38% (*n* = 102), 59% (*n* = 161), and 39% (*n* = 103), respectively.

#### Recidivism

We obtained post-release recidivism rates for a smaller subsample of the juvenile and adult units (*n* = 116) based on police records. Six cases with a follow-up lower than 6 months were excluded, *n* = 110 offenders remained in the analyses with an average time at risk of *M* = 22.34 months (*SD* = 7.72, Range = 7.92–34.83). These records capture whether the police accused a person being a strong suspect of a crime. Therefore, they have a lower threshold compared to convictions of a criminal record. In addition, the records only cover crime accusations in Berlin, but not for the whole Germany. The research group coded whether a participant was accused of a non-violent crime (e.g., thievery, drug offenses, violations of instructions, or driving without a license), a violent crime (e.g., robbery, assault, or manslaughter), and a sexual crime (e.g., sexual abuse or rape). Recidivism rates were 38% (*n* = 42) for non-violent and 13% (*n* = 14) for violent recidivism. Due to the low recidivism rate of 4% (*n* = 4) sexual recidivism was excluded from further analyses.

### Data Analysis

Statistical analysis was performed using SPSS 22 for Windows. Sample size was acceptable to perform factor analysis (44). Beforehand, parallel analysis (45) was employed to determine the appropriate number of factors to extract. The procedure is based on Monte Carlo simulations and has been proven to be accurate in determining the threshold for significant factors (46). The items were then subjected to principal axis factor analysis with oblique rotation. Common factor procedures with intercorrelated factors are preferably used to identify psychological meaningful constructs (47). Items were retained when primary factor loadings exceeded.32 and cross-loading differences were < 0.20 (48). Bivariate Pearson correlations were calculated to examine associations with risk assessment instruments. Predictive validity of the SWAP-RS was examined using receiver operating characteristic (ROC) analysis. The use of the area under the curve (AUC) is the preferred measure of predictive accuracy in forensic assessment, and AUCs of 0.56, 0.64, 0.71 indicate small, moderate, and large effects, respectively (49). Finally, hierarchical block-wise logistic regressions were used to investigate incremental validity of the SWAP-RS. Unless otherwise stated alpha level was set at *p* < 0.05.

## Results

### Factor Analysis

Parallel analysis indicated that three factors should be retained. The 40 items were subjected to principal axis factoring with oblique rotation. Both the Kaiser-Meyer-Olkin measure (KMO = 0.93; values for individual items ranged from 0.81 to 0.97) and Bartlett's test of sphericity (χ2_(780)_ = 7077.88, *p* < 0.001) verified sampling adequacy for the analysis. The three factors accounted for a substantial amount of variance (54.55%). Table 1 presents factor loadings after rotation, eigenvalues, and percentage of variance for each factor. All the 40 items could be retained. The first factor accounted for 32.07% of the total variance and seems to represent all the items of the SWAP-200 factors psychopathy, hostility, narcissism, and emotional dysregulation. Noteworthy, the item “lacks social skills,” which represents a feature of schizoid orientation according to the SWAP-200, showed highest loadings on the first factor. All these items are considered problematic behaviors that are directed toward the external environment. Therefore, the factor was labeled “Externalizing Prison Behavior” (EPB). The second factor accounted for 12.16% of total variance, corresponds to all the psychological health items of the SWAP-200, and was therefore labeled “Adaptive Prison Behavior” (APB). We defined adaptive behavior as a collection of social and emotional coping strategies to function in the prison environment, however, we do not refer to the extensive research field of mental retardation. Finally, the third factor accounted for 7.25% of the total variance and was comprised of the items assigned to the dysphoria and schizoid orientation factors of the SWAP-200. Accordingly, it was labeled “Internalizing Prison Behavior” (IPB).

**Table 1:** Summary of factor analysis of the SWAP-RS items; factor loadings after rotation (*N* = 272).

	**I**	**II**	**III**
Seeks to be the center of attention	0.85	
Has an exaggerated sense of self-importance	0.84	
Appears to feel privileged and entitled, expects preferential treatment	0.82	
Tends to be arrogant, haughty, or dismissive	0.79	
Seems to treat others primarily as an audience to witness own importance and brilliance	0.78	
Tends to hold grudges, may dwell on insults or slights for long periods	0.78	
Takes advantage of others, is out for number one, has minimal investment in moral values	0.78	
Tends to express intense and inappropriate anger that is out of proportion to the situation at hand	0.75	
Tends to be critical of others	0.75	
Expresses emotion in exaggerated and theatrical ways	0.75	
Tends to be hostile	0.74	
Emotions tend to change rapidly and unpredictably	0.71	
Tends to be deceitful, tends to lie or mislead	0.70	
Appears to experience no remorse for harm or injury caused to others	0.68	
Tends to become irrational when strong emotions are stirred up	0.65		0.33
Tends to show reckless disregard for the rights, property, or safety of others	0.64	
Tends to assume that others have bad and malevolent intentions	0.61	
Emotions tend to spiral out of control, leading to extremes of anxiety, sadness, rage, excitement, etc.	0.60		0.33
Lacks social skills; tends to be socially awkward or inappropriate	0.56		0.34
Is unable to soothe or comfort self when distressed, requires involvement of another person to help regulate affect	0.54	
Tends to be unreliable and irresponsible	0.46	
Is able to find meaning and satisfaction in the pursuit of long-term goals and ambitions		0.74
Enjoys challenges, takes pleasure in accomplishing things		0.73
Is capable of sustaining a meaningful love relationship characterized by genuine intimacy and caring		0.68
Is capable of hearing information that is emotionally threatening		0.65
Is empathic, is sensitive and responsive to other peoples' needs and feelings		0.64
Is able to use his talents, abilities, and energy effectively and productively		0.63
Tends to be conscientious and responsible		0.63
Appears comfortable and at ease in social situations		0.49
Appreciates and responds to humor		0.49
Is able to assert himself effectively and appropriately when necessary		0.36
Tends to feel he is inadequate, inferior, or a failure			0.83
Tends to feel empty or bored			0.74
Appears to find little or no pleasure, satisfaction, or enjoyment in life's activities			0.74
Tends to feel life has no meaning			0.74
Tends to feel like an outcast or outsider, feels as if he does not truly belong			0.73
Tends to feel listless, fatigued, or lacking in energy			0.53
Lacks close friendships and relationships			0.51
Tends to be shy or reserved in social situations.			0.51
Appears to have little need for human company or contact, is genuinely indifferent to the presence of others			0.45
Eigenvalue	12.83	4.87	2.90
% of variance	32.07	12.16	7.25

*Factor loadings <0.32 are not displayed*.

Internal consistencies (as indicated by Cronbach's alpha) of the factors EPB, APB, and IPB were 0.96, 0.87, and 0.89, respectively. Nunnally (50) suggested internal consistencies of 0.90 as the minimum in applied settings, which was (almost) achieved by the three factors. Corrected item-total correlations of the factors ranged from 0.54–0.79, 0.41–0.71, to 0.47–0.76, respectively. Interrater reliability of the ratings was examined in a subsample randomly selected from the juvenile unit. Two prison officers independently rated *n* = 23 inmates within *M* = 1.02 months (*SD* = 0.81; Range = 0–2.60). Significant agreement was found for the factor EPB, single measure intraclass correlation coefficient (ICC) was.48, *p* < 0.01, 95% confidence interval (95% CI) [0.09, 0.74], and APB, ICC = 0.55, *p* < 0.01, 95% CI [0.18, 0.78], indicating moderate rater agreement (51). However, this was not the case for IPB, ICC = 0.33, *p* = 0.059, 95% CI [0.09, 0.65]. The SWAP-RS factors showed moderate intercorrelations. As expected, APB was negatively associated with EPB and IPB, and EPB was positively associated with IPB. Table 2 summarizes the psychometric properties of the SWAP-RS factors. Unit-weighted mean scores were calculated for each factor for further analyses.

**Table 2:** Summary of psychometric properties of the SWAP-RS factors.

	**EPB**	**APB**	**IPB**
Externalizing Prison Behavior (EPB)	–	
Adaptive Prison Behavior (APB)	−0.25**	–
Internalizing Prison Behavior (IPB)	0.45**	−0.39**	–
Mean (*SD*)	1.11 (0.84)	1.70 (0.68)	1.24 (0.75)
Range	0–3.52	0–3.40	0–3.90
# items	21	10	9
Internal consistency^1^	0.96	0.87	0.89
Interrater reliability^2^	0.48**	0.55**	0.33

* *p < 0.05*,

** p < 0.01;

1 Crohnbach's alpha;

2 *Intraclass correlation coefficient*.

### Construct Validity

To examine construct validity correlations were calculated between the SWAP-RS factors and a risk measure for general recidivism (LSI-R), a measure for violence risk assessment (HCR-20), and a rating scale for the clinical construct of psychopathy (PCL-R; see Table 3). As hypothesized, the convergent validity of EPB was evidenced by small significant relationships with the total scores of the LSI-R (*r* = 0.23, *p* < 0.01), HCR-20 (*r* = 0.23, *p* < 0.01), PCL-R (*r* = 0.24, *p* < 0.01). A more differentiated analysis of the LSI-R revealed significant associations between EPB and the scales Criminal History (*r* = 0.13, *p* < 0.05), Education and Employment (*r* = 0.13, *p* < 0.05), Financial (*r* = 0.17, *p* < 0.01), Leisure and Recreation (*r* = 0.12, *p* < 0.05), Companions (*r* = 0.12, *p* < 0.05), Emotional and Personal (*r* = 0.14, *p* < 0.05), and Attitudes and Orientation (*r* = 0.22, *p* < 0.001). Correlations between EPB and HCR-20 subscales were highest for the Clinical subscale (*r* = 0.24, *p* < 0.01). Regarding the PCL-R, EPB showed stronger correlations with Factor 2 (*r* = 0.25, *p* < 0.01) than with Factor 1 (*r* = 0.16, *p* < 0.05). In contrast, the APB scale did not show any relationships with the total scores of the risk measures. However, as expected, all the (non-significant) relationships had a negative trend. Only PCL-R Factor 2 was negatively related to APB (*r* = −0.14, *p* < 0.05). The IPB scale did not show any significant relationships with the total scores. However, on a scale level IPB was associated with the LSI-R subscales Financial (*r* = 0.17, *p* < 0.01), Family and Marital (*r* = 0.20, *p* < 0.01), and Emotional and Personal (*r* = 0.24, *p* < 0.001). Furthermore, there was a small positive association between IPB and the risk management subscale of the HCR-20 (*r* = 0.14, *p* < 0.05)

**Table 3:** Pearson correlations between SWAP-RS factors and actuarial risk measures.

	**LSI-R**	**HCR-20**				**PCL-R**		
	**Total**	**Total**	**Historical**	**Clinical**	**Risk**	**Total**	**Factor 1**	**Factor 2**
EPB	0.23**	0.23**	0.18**	0.24**	0.15*	0.24**	0.16*	0.25**
APB	−0.08	−0.11	−0.08	−0.09	−0.08	−0.10	−0.05	–0.14*
IPB	0.05	0.10	0.05	0.10	0.14*	0.05	0.08	0.04

* *p < 0.05*,

** *p < 0.01*.

*LSI-R, Level of Service Inventory—Revised; HCR-20, Historical-Clinical-Risk Scheme; PCL-R, Psychopathy Checklist—Revised. EPB, Externalizing Prison Behavior; ABP, Adaptive Prison Behavior; IPB, Internalizing Prison Behavior*.

### Predictive Validity

The area under the curve (AUC) values of the SWAP-RS factors are presented in Table 4. EPB was predictive of violent recidivism (0.78), as well as violent and non-violent institutional misconduct (both 0.62). APB and IPB were significant predictors of granted privileges (0.64 and 0.61). Importantly, the correlation between IPB and granted privilege was negative (*r* = −0.18, *p* < 0.01), therefore the value of the state variable in the ROC analysis was set to 0. This means that inmates with high ratings of internalizing behavior were less likely to receive privileges. Finally, IPB was a significant predictor of violent recidivism (0.69). In comparison, for example, the AUCs of the LSI-R for violent misconduct, non-violent misconduct, violent and non-violent recidivism were 0.63 (95% CI [0.56, 0.70]),0.64 (95% CI [0.57, 0.71]),0.71 (95% CI [0.58, 0.85]), and 0.66 (95% CI [57, 0.78]), respectively. AUCs of the HCR-20 and PCL-R were predominantly significant but somewhat lower with values between 0.48–0.60 and 0.50–0.56, respectively.

**Table 4:** Predictive accuracy of the SWAP-RS factors for institutional behavior and recidivism (AUC).

	**Misconduct**		**Conduct**	**Recidivism**	
	**Violent**	**Non-violent**	**Privilege**	**Violent**	**Non-violent**
EPB	0.62** **[0.55,0.69]**	0.62** **[.55,0.69]**	0.46 [0.39,0.53]	0.78** **[0.66,0.91]**	0.56 [0.44,0.67]
APB	0.44 [0.37,0.51]	0.51 [.44,0.58]	0.64*** **[0.57,0.70]**	0.37 [0.24,0.51]	0.52 [0.41,0.63]
IPB	0.51 [0.44,0.59]	0.49 [.42,0.56]	0.61** **[0.54,0.68]**^a^	0.69** **[0.56,0.83]**	0.45 [0.34,0.56]

* *p < 0.05*,

** *p < 0.01*,

*** *p < 0.001*.

a *The association was negative and the state variable set to 0. AUC, Area under the curve; 95% confidence interval in brackets. EPB, Externalizing Prison Behavior; ABP, Adaptive Prison Behavior; IPB, Internalizing Prison Behavior*.

### Incremental Validity

To test incremental validity of the SWAP-RS hierarchical block-wise logistic regression was used. For each regression analysis, the LSI-R, HCR-20, and PCL-R were entered into the first block of the model. To test which SWAP-RS factor added incremental validity to the risk measures, if any, a forward-method (i.e., likelihood ratio method) was employed for block 2. In block 1 of the hierarchical regression model for violent misconduct (χ2_(3)_ = 15.76, *p* < 0.01), accounting for 8% (Nagelkerke) of the variance, the LSI-R was a significant predictor (*B* = 0.06, *p* < 0.05; see Table 5). In block 2, EPB was the only predictor (*B* = 0.50, *p* < 0.01) to add incremental validity to the model (χ2_(4)_ = 25.52, *p* < 0.001). The latter model accounted for 12% of the variance, which is a significant increase from block 1 (χ2_(1)_ = 9.77, *p* < 0.01). The AUC for block 1 was 0.64 (95% CI [0.57, 0.71]), and 0.67 (95% CI [0.61, 0.74] after adding EPB in block 2.

**Table 5:** Incremental validity of the SWAP-RS factors in relation to actuarial risk measures predicting institutional behavior.

	**Violent misconduct**^a^		**Non-violent misconduct**^b^		**Privilege**^c^	
	**B (SE)**	**Exp b [95% CI]**	**B (SE)**	**Exp b [95% CI]**	**B (SE)**	**Exp b [95% CI]**
**BLOCK 1**						
Constant	−2.67 (0.56)		−0.96 (0.53)		−0.89 (0.63)
LSI-R	0.05* (0.03)	1.05 [1.00, 1.11]	0.08** (0.03)	1.09 [1.03, 1.15]	0.01 (0.05)	1.01 [0.96, 1.06]
HCR-20	0.06 (0.05)	1.06 [0.97, 1.16]	−0.02 (0.05)	0.98 [0.89, 1.07]	−0.05 (0.05)	0.95 [0.87, 1.04]
PCL-R	−0.05 (0.21)	0.95 [0.88, 1.02]	−0.02 (0.04)	0.98 [0.92, 1.05]	−0.01 (0.04)	0.99 [0.92, 1.06]
**BLOCK 2**						
EPB	0.50** (0.16)	1.65 [1.20, 2.28]	0.62** (0.19)	1.86 [1.28, 2.71]	
APB					0.72*** (0.20)	2.05 [1.48, 3.06]
IPB			–0.50* (0.20)	0.61 [0.41, 0.90]	

a *R2 = 0.12 (Nagelkerke). Model χ2_(4)_ = 25.52, p < 0.001. Hosmer-Lemeshow test χ2_(8)_ = 2.04, ns*.

b *R2 = 0.14 (Nagelkerke). Model χ2_(3)_ = 29.68, p < 0.001. Hosmer-Lemeshow test χ2_(8)_ = 6.94, ns*.

c *R2 = 0.10 (Nagelkerke). Model χ2_(3)_ = 19.65, p < 0.001. Hosmer-Lemeshow test χ2_(8)_ = 13.16, ns*.

* *p < 0.05*;

** *p < 0.01*;

*** *p < 0.001. LSI-R, Level of Service Inventory—Revised; HCR-20, Historical-Clinical-Risk Scheme; PCL-R, Psychopathy Checklist—Revised. EPB, Externalizing Prison Behavior; ABP, Adaptive Prison Behavior; IPB, Internalizing Prison Behavior*.

The logistic regression model predicting non-violent misconduct was found to be significant in block 1 (χ2_(3)_ = 17.28, *p* < 0.01), accounting for 8% (Nagelkerke) of the variance (see Table 5). Again, the LSI-R was a significant predictor (*B* = 0.09, *p* < 0.001). In block 2, EPB (*B* = 0.62, *p* < 0.01) and IPB (*B* = −0.50, *p* < 0.05) were found to be significant predictors to the model (χ2_(5)_ = 29.68, *p* < 0.001). The final model accounted for 14% of the variance, which is a significant increase (χ2_(2)_ = 12.40, *p* < 0.01). The AUC for block 1 was 0.62 (95% CI [0.55, 0.69]), and 0.67 (95% CI [0.61, 0.74] after including EPB and IPB (block 2). An additional regression analysis was carried out to investigate a possible interaction between EBP and IPB. We ran the same model adding an interaction term (EBPxIPB) in block 2, however, the interaction term did not add incrementally to the model.

The logistic regression model predicting whether an inmate was granted a privilege was not significant in block 1 (χ2_(3)_ = 6.37, *p* = 0.10; see Table 5). After adding the SWAP-RS factors in block 2, a significant model was produced (χ2_(4)_ = 19.65, *p* < 0.01), accounting for 10% of the variance, with APB being the only single significant predictor (*B* = 0.72, *p* < 0.01). The increase was found to be significant (χ2_(1)_ = 13.28, *p* < 0.001). The AUC for the block 1 model was 0.59 (95% CI [0.52, 0.66]) and 0.66 (95% CI [0.35, 0.49]) after including APB.

The logistic regression model predicting post-release violent recidivism was found to be significant in block 1 (χ2_(3)_ = 13.60, *p* < 0.01), accounting for 21% (Nagelkerke) of the variance (see Table 6). The LSI-R (*B* = 0.22, *p* < 0.01) and HCR-20 (*B* = −0.31, *p* < 0.05) were significant predictors, however, the negative sign of the HCR-20 was unexpected^3^. In block 2, again EPB (*B* = 1.63, *p* < 0.01) added incrementally to the model (χ2_(4)_ = 27.49, *p* < 0.001), which was a significant increase (χ2_(1)_ = 13.90, *p* < 0.001). The AUC of the block 1 model was 0.78 (95% CI [0.65, 0.91]) and 0.89 (95% CI [0.82, 0.96]) after including EPB. Finally, the regression model for non-violent post-release recidivism was found to be significant in block 1 (χ2_(3)_ = 12.87, *p* < 0.01), accounting for 15% of the variance (see Table 6). Again, the LSI-R (*B* = 0.14, *p* < 0.01) was the only significant predictor in the model. Block 2 revealed that none of the SWAP-RS factors were found to be significant predictors. The AUC of the block 1 model was 0.70 (95% CI [0.60, 0.81]).

**Table 6:** Incremental validity of the SWAP-RS factors in relation to actuarial risk measures predicting recidivism.

	**Violent recidivism**^a^		**Non-violent recidivism**^b^	
	**B (SE)**	**Exp b [95% CI]**	**B (SE)**	**Exp b [95% CI]**
**BLOCK 1**				
Constant	−5.23** (1.66)		−2.39** (0.92)
LSI-R	0.23** (0.08)	1.25 [1.08, 1.45]	0.14** (0.04)	1.15 [1.06, 1.26]
HCR-20	−0.37* (0.16)	0.69 [0.51, 0.96]	−0.07 (0.07)	0.93 [0.82, 1.07]
PCL-R	0.11 (0.12)	1.12 [0.89, 1.41]	−0.03 (0.06)	0.97 [0.85, 1.10]
**BLOCK 2**				
EPB	1.63** (0.47)	5.09 [2.01, 12.87]	
APB			
IPB			

a *R2 = 0.42 (Nagelkerke). Model χ2_(4)_ = 27.49, p < 0.001. Hosmer-Lemeshow test χ2_(8)_ = 7.97, ns*.

b *R2 = 0.14 (Nagelkerke). Model χ2_(3)_ = 12.36, p < 0.01. Hosmer-Lemeshow test χ2_(8)_ = 11.06, ns*.

* *p < 0.05*;

** *p < 0.01*;

*** *p < 0.001*.

*LSI-R, Level of Service Inventory—Revised; HCR-20, Historical-Clinical-Risk Scheme; PCL-R, Psychopathy Checklist—Revised; EPB, Externalizing Prison Behavior; ABP, Adaptive Prison Behavior; IPB, Internalizing Prison Behavior*.

## Discussion

The purpose of this study was to investigate the applicability and validity (construct, predictive, and incremental) of a behavior rating scale assessed by prison officers, the SWAP rating scale (SWAP-RS). The first part addressed the construct validity of the SWAP-RS. The leading questions were (a) do prison officers observe behaviors that map onto psychologically meaningful factors, and (b) do these observations correspond to standardized risk measures. In the second part we examined predictive validity of the factors thus identified. Here, the questions of interest were (a) are ratings of observed prison behavior useful for predicting institutional (mis-) conduct and recidivism, and (b) do they incrementally improve predictive accuracy of established risk assessment procedures.

Based on empirical and theoretical considerations, a shortened set of SWAP-200 items was selected to assess prison officers' observations of inmate behavior. Factor analysis suggested a psychologically meaningful three-factor solution. The first factor (Externalizing Prison Behavior [EPB]) appears to represent behavioral characteristics related to psychopathy, hostility, narcissism, and emotional dysregulation. The second factor (Adaptive Prison Behavior [APD]) seems to represent characteristics of psychological health and resources. Finally, the third factor (Internalizing Prison Behavior [IPB]) seems to represent characteristics related to dysphoria and schizoid orientation. The factor structure strongly resembles higher-order dimensions referring to externalizing and internalizing behavior (30). For example, Westen and colleagues found that psychopathic and narcissistic characteristics form an externalizing dimension, and dysphoria and schizoid orientation an internalizing dimension. Additionally, the psychological health items were represented on a distinct dimension termed adaptive personality strengths. Krueger et al. (53) also support the notion of two broad dimensions positing externalizing and internalizing features. Krueger et al. (53) stated that externalizing behavior is linked to a lack of constraint (e.g., to engage in risky behavior, to act on impulse, to endorse non-traditional values), and internalizing to negative emotionality (e.g., to experience anxiety, alienation from others). Furthermore, externalizing behavior is associated with substance dependence and antisocial behavior, whereas internalizing behavior is associated with anxiety disorders and depression [e.g., (54)]. Noteworthy, the first factor appears to capture a broad range of socially aversive behaviors (55). Only recently, a growing body of research on the so-called “Dark Triad,” a constellation of psychopathic, narcissistic, and machiavellistic personality features, has highlighted the empirical overlap of these constructs in non-pathological samples (56). Although research indicates that the Dark Triad constructs are conceptually distinct, they share characteristics such as callousness, hostility, and impulsivity (57), and were found to be associated with aggressive and criminal behavior [for overview see Furnham (58)]. The EPB factor seems to tap into some features of the Dark Triad.

The psychometric properties of the SWAP-RS were generally satisfactory. Internal consistencies of the scales were appropriate for applied settings (50). In contrast, the results of interrater reliability were less strong. Whereas interrater reliability of the factors EPB and APB was moderate (51), the prison officers showed less agreement about internalizing behaviors. One explanation may be that behaviors related to the EPB and APB factors are rather directed toward the external environment, whereas items of the IPB factor are directed toward the “self” and thus harder to be externally identified. Cooke (19) further argued that prison officers may be more experienced observers of disruptive behavior because it is closely related to safety concerns and suggested to train prison officers. Training may not only lead to improved agreement, but also deepen the awareness, knowledge and acceptance of certain behaviors. Noteworthy, many prison officers commented positively on the SWAP-RS. Amongst other things, they stated that the assessment has led to more intense engagement with the prisoners and their behavior.

Differential associations with established risk assessment measures further evidenced construct validity of the SWAP-RS. As expected, the EPB factor was significantly associated with the LSI-R, HCR-R, and the PCL-R, whereas APB and IPB were almost unrelated to the instruments. The correlations indicated that the EPB factor may capture behavioral characteristics that are associated with antisocial orientation (37). For example, EPB was significantly related to the attitudes and orientation subscale of the LSI-R. Furthermore, items such as “appears to experience no remorse,” “takes advantage of others,” and “has an exaggerated sense of self-importance” are reminiscent of characteristics of the construct of psychopathy (36). Accordingly, the results indicated that the EPB is correlated with the PCL-R. Interestingly, EPB ratings were stronger associated with the lifestyle antisociality dimension of the PCL-R. This may correspond to the notion that Factor 2 of the PCL-R highlights the behavioral correlates of psychopathy (36). Some research suggests that Factor 2 of the PCL-R outperforms Factor 1 in predicting institutional misconduct and recidivism [e.g., (59)]. In line with Cooke (19) these findings indicate that prison officers may be able to assess behavioral characteristics related to the psychopathy construct. Similarly, the significant associations between EPB and HCR-20, and in particular the clinical subscale show that items such as “emotions tend to change rapidly and unpredictably” and “emotions tend to spiral out of control” may tap the construct of impulsivity, which is among the strongest individual predictor of recidivism [e.g., (11, 60)].

The APB factor consists of items such as “tends to be conscientious and responsible” and “enjoys challenges and takes pleasure in accomplishing things” and refers to psychological strengths and resources (34). As expected, we found no associations between APB and the total scores of the risk assessment measures. Therefore, these behaviors may not constitute a risk factor *per se*. In contrast, they may rather capture individual skills and coping strategies that are needed to deal with the psychological effects of imprisonment (61). Finally, the IPB factor consists of items such as “tends to feel he is inadequate” and “tends to feel empty or bored.” Correlational analyses indicated rather weak associations between internalizing behavior and the risk measures. However, construct validity of the factor was evidenced by meaningful associations with the emotional subscale of the LSI-R and the R-scale of the HCR-20. For example, the LSI-R subscale assesses an individual's ability to respond to life stressors and psychological signs of anxiety and depression (37).

Prison officers' ratings of inmate behavior were not only predictive of misconduct and conduct within the prison setting, but also of recidivism after release. Foremost, ratings of externalizing behaviors were predictive of violent and non-violent misconduct and violent recidivism. Predictive accuracy was moderate for both criteria of misconduct in prison and large for violent recidivism after release. Notably, prison officers' ratings of externalizing behaviors predicted violent recidivism better than the LSI-R. These findings further indicate that the EPB factor taps risk-relevant behaviors. Comparable results were provided by previous research on the predictive validity of behavioral ratings by staff (20, 26, 28).

The APB factor significantly predicted whether an inmate was granted privileges or not. This finding emphasizes that it may be beneficial to assess behavioral strengths and resources in offender rehabilitation. Recent research suggested that the quality of release planning added incremental validity to the prediction of recidivism over and above standardized risk measures (62). In Germany, privileges (i.e., day release, outside employment, or minimum-security confinement) are acknowledged as central methods for treatment and prisoner reentry. Accordingly, Suhling and Rehder (63) reported that sexual offenders in minimum-security confinement have lower rates of recidivism. Therefore, it may be possible that adaptive behavior in prison has a moderator effect on future recidivism (i.e., inmates showing high levels of adaptive behavior in prison are more likely to receive privileges, which in turn has an effect on future recidivism). Clearly, future research is needed to investigate this relationship. Finally, the IPB factor was also predictive of violent recidivism. This corresponds to a large body of research suggesting that emotional distress and psychopathology (e.g., depression) are considered minor risk factors for criminality (37). Interestingly, inmates with high ratings of internalizing behaviors were less likely to receive any kind of granted privileges.

While the above findings support the predictive validity of the SWAP-RS, it would be inappropriate to use prison officers' observations alone for risk assessment purposes. As mentioned before, the rating scale is intended to be a supplement to established risk scales. To our knowledge, the present study is the first to investigate the incremental validity of a behavior rating scale assessed by prison officers. The SWAP-RS significantly improved prediction beyond standardized risk assessment instruments. Specifically, prison officers' ratings of externalizing behavior added incremental validity to the LSI-R for the prediction of violent misconduct and violent recidivism, whereas both EPB and IPB added incrementally to the LSI-R for non-violent misconduct. Especially for violent recidivism, the inclusion of the EPB factor lead to a substantial increase in predictive accuracy. These findings suggest that observations of current behavior provide information for the prediction of violent misconduct and violent recidivism, which does not seem to be captured by established risk assessment instruments. This emphasizes the importance of including measures of current risk-relevant behavior into risk assessment procedures. Noteworthy, whereas higher levels of externalizing behavior were positively associated with non-violent misconduct, the model revealed negative associations for internalizing behavior. That may imply that inmates with high ratings of internalizing and low ratings of externalizing behaviors are less likely to show misconduct in prison. Cooke (20) reported similar findings for the prediction of institutional misconduct, suggesting improved prediction after combining the Antiauthority and Dull-Confused scales of the PRBS. However, such an interaction could not be confirmed in the present study.

Notably, prison officers' ratings on adaptive behavior remained the only significant predictor of granted privileges. This is somewhat surprising since prior research has shown that, for example, the LSI-R is a robust predictor of security-level placement in prison (38). An explanation may be that the outcome variable in the present study included too many kinds of privileges or the sample was too heterogeneous. For example, inmates under preventive detention receive usually less privileges and are therefore hardly comparable with inmates of the two correctional treatment units.

Several limitations of the present study merit consideration. The inmates were assessed by many prison officers. Therefore, a large variance is to be expected, which is particularly problematic given the weak to moderate rater agreement. To reduce variability brief training sessions are suggested in future studies. In addition, it seems important to consider the influence of prison officers' individual factors (e.g., work motivation and attitude) and personal closeness to inmates as a source of variation. In a similar manner, potential rater biases (e.g., leniency or severity effects) require investigation. The sample of the present study was quite heterogeneous regarding age and offense type. For example, the relationship between age and externalizing behavior (e.g., aggression) in prison is a consistent finding in the literature [e.g., (61)]. Therefore, future research should also investigate whether institutional factors affect prison officers' ratings (e.g., prison officers at a juvenile unit may be more habituated to aggressive behaviors and therefore have different rating thresholds). The factors showed meaningful associations with the risk assessment measures albeit the relationships were rather small. Therefore, farther construct validation with risk-related measures (e.g., self-report) is desirable. Finally, it is important to mention that the current approach differs from the offense paralleling framework (6). One specific assumption is that offense paralleling behavior must be understood in terms of functionality, not simply appearance. For example, reckless behavior in prison may be considered as an indicator of a risk-related propensity. However, the behavior may only be triggered by the environment (e.g., as an adjustment strategy in prison) and therefore may not be indicative of such a propensity. Consequently, the framework requires a complex process of analysis that could not be realized in the current study (8).

In conclusion, there is consensus that forensic risk assessment benefits from including a variety of information, inter alia, crime scene analysis (64) and standardized risk measures which incorporate static and dynamic risk factors [e.g., (3)]. The assessment of current behavior, however, was predominantly disregarded for risk assessment purposes (65). In line with previous research [e.g., (20)], the present study has shown that the supplemental use of prison officers' ratings of inmate behavior can improve risk assessment. Although the validity of the EPB factor was most convincing, it may be advisable to assess various characteristics of prison behavior to fully understand behavioral changes (6). Pragmatically, the SWAP-RS allows prison officers to systematically rate inmates' behavior in a quick and reliable manner and can be easily implemented into regular case management routines. We conclude that prison officers' observations, if assessed systematically, can be a valuable complement for treatment evaluation and risk assessment.

## Author Contributions

JH, RL, and K-PD contributed conception and design of the study. JH organized the database and performed the statistical analysis and wrote the first draft of the manuscript. RL wrote parts of the manuscript and revised the first draft. All authors contributed to manuscript revision, read, and approved the submitted version.

### Conflict of Interest Statement

The authors declare that the research was conducted in the absence of any commercial or financial relationships that could be construed as a potential conflict of interest.

## Footnotes

^1^Eight offenders served a life sentence. In line with the International Criminal Court in the Hague, Netherlands, life sentences were generally coded as 25 years. In Germany, in 2015 *n* = 59 offenders serving a life sentence were released after *M* = 19.3 years (Range = 14.8 – 49.8).

^2^The authors termed this factor psychopathy and described it as a combination of antisocial personality disorder and psychopathy characteristics (34). Importantly, the SWAP-200 factor psychopathy is not eligible to assess the clinical construct of psychopathy (36).

^3^Since there was a significant correlation between violent recidivism and the LSI-R (*r* = 0.21, *p* < 0.05), but not with the HCR-20 (*r* = −0.05, *p* = 0.56), and a strong positive relationship between the LSI-R and the HCR-20 (*r* = 0.70, *p* < 0.001), the HCR-20 appears to be a suppressor in the model [i.e., it removes irrelevant variance of the LSI-R; (52)]. A similar regression model was produced after removing the HCR-20 (χ2_(3)_ = 20.33, *p* < 0.001). As expected, the explained variance (Nagelkerke = 0.32) was somewhat lower, but both LSI-R (*B* = 0.14, *p* < 0.05) and EPB (*B* = 1.52, *p* < 0.01) remained significant predictors of violent recidivism.

## References

[1] Blackburn R Renwick SJ Rating scales for measuring the interpersonal circle in forensic psychiatric patients. Psychol. Assess. (1996) 8:76–84. 10.1037/1040-3590.8.1.76

[2] Clark DA Fisher MJ McDougall C A new methodology for assessing the level of risk in incarcerated offenders. Br. J. Criminol. (1993) 33:436–48. 10.1093/oxfordjournals.bjc.a048335

[3] Lehmann RJB Fernandez Y Helmus L-M Strengths of actuarial risk assessment. In: Laws DR O‘Donohue W editors, Treatment of Sex Offenders. Heidelberg: Springer (2016). p. 45–81.

[4] Mann RE Hanson RK Thornton D. Assessing risk for sexual recidivism: some proposals on the nature of psychologically meaningful risk factors. Sexual Abuse (2010) 22:191–217. 10.1177/1079063210366039 20363981

[5] Shoda Y A unified framework for the study of behavioral consistency: bridging person × situation interaction and the consistency paradox. Eur. J. Pers. (1999) 13:361–87.

[6] Jones LF Offence paralleling behavior (OPB) as a framework for assessment and interventions with offenders. In: Needs A Towl G, editors, Applying Psychology to Forensic Practice, Oxford: BPS-Blackwell (2004). p. 34–63.

[7] Mann RE Thornton D Wakama S Dyson M Atkinson D Applying the concept of offence paralleling behaviour to sex offender assessment in secure settings. In: Daffern M Jones L Shine J, editors, Offence Paralleling Behavior: A Case Formulation Approach to Offender Assessment and Intervention. Oxford: Wiley-Blackwell (2010). p. 121–36.

[8] Atkinson DF Mann RE Prison officers as observers of offence paralleling behaviours: an untapped resource? J Forensic Psychiatry Psychol. (2012) 23:139–55. 10.1080/14789949.2012.668209

[9] Merrell KW Behavioral, Social, and Emotional Assessment of Children and Adolescents (2nd.). Mahwah, NJ: Lawrence-Erlbaum (2002).

[10] Barriga AQ Landau JR Stinson BL Liau AK Gibbs JC Cognitive distortion and problem behaviors in adolescents. Crim. Justice Behav. (2000) 27:36–56. 10.1177/0093854800027001003

[11] Gendreau P Little T Goggin CE A meta-analysis of the predictors of adult offender recidivism: what works!. Criminology (1996) 34:575–607. 10.1111/j.1745-9125.1996.tb01220.x

[12] Milton J McCartney M Duggan C Evans C Collins M McCarthy L Beauty in the eye of the beholder? How high security hospital psychopathically-disordered patients rate their own interpersonal behaviour. J Forensic Psychiatry Psychol. (2005) 16:552–65. 10.1080/14789940500098194

[13] Westen D Weinberger J. When clinical description becomes statistical prediction. Am Psychol. (2004) 59:595–613. 10.1037/0003-066X.59.7.595 15491255

[14] McConaughy SH Ritter DR Best practices in multidimensional assessment of emotional or behavioral disorders. In Thomas A Grimes J. editors. Best practices in school psychology. Washington, DC: NASP (2002). p. 865–873.

[15] Dvoskin JA Spiers EM. On the role of correctional officers in prison mental health. Psychiatr Quart. (2004) 75:41–59. Available online at: https://link.springer.com/article/10.1023/B:PSAQ.0000007560.09475.a0 14992302 10.1023/b:psaq.0000007560.09475.a0

[16] Quay HO Standards for Adult Correctional Institutions. Washington, DC: Federal Bureau of Prisons (1983).

[17] Levinson RB Developments in the classification process: quay's AIMS approach. Crim. Justice Behav. (1988) 15:24–38. 10.1177/0093854888015001004

[18] Cooke DJ Walker A Gardiner W Behavioral disturbance in Barlinnie Prison. Prison Serv J. (1990) 80:2–8.

[19] Cooke DJ The development of the prison behavior rating scale. Crim. Justice Behav. (1998) 25:482–506. 10.1177/0093854898025004005

[20] Cooke DJ Predicting offending in prison: the predictive validity of the Prison Behaviour Rating Scales. Legal Criminol Psychol. (1996) 1:65–82. 10.1111/j.2044-8333.1996.tb00307.x

[21] Blackburn R Criminality and the interpersonal circle in mentally disordered offenders. Crim. Justice Behav. (1998) 25:155–76. 10.1177/0093854898025002001

[22] Blackburn R Glasgow DV Manual for the Chart of Interpersonal Reactions in Closed Living Environments (CIRCLE). Unpublished manuscript, University of Liverpool (2006).

[23] Doyle M Dolan M. Evaluating the validity of anger regulation problems, interpersonal style, and disturbed mental state for predicting inpatient violence. Behav Sci Law (2006) 24:783–98. 10.1002/bsl.739 17171766

[24] Daffern M Tonkin M Howells K Krishnan G Ijomah G Milton J The impact of interpersonal style and perceived coercion on aggression and self-harm in personality-disordered patients admitted to a secure psychiatric hospital. J Forensic Psychiatry Psychol. (2010) 21:426–45. 10.1080/14789940903505951

[25] Vernham Z Tapp J Moore E. Observer ratings of interpersonal behavior as predictors of aggression and self-harm in a high-security sample of male forensic inpatients. J Interpers Violence (2016) 31:1597–617. 10.1177/0886260515569060 25646165

[26] Dolan M Blackburn R Interpersonal factors as predictors of disciplinary infractions in incarcerated personality disordered offenders. Pers Individ Dif. (2006) 40:897–907. 10.1016/j.paid.2005.10.003

[27] Marin-Avellan LE McGauley G Campbell C Fonagy P. Using the SWAP-200 in a personality-disordered forensic population: is it valid, reliable and useful? Crim Behav Mental Health (2005) 15:28–45. 10.1002/cbm.35 16470497

[28] Hausam J Lehmann RJ Dahle KP Diagnostischer Nutzen einer strukturierten Erfassung von Verhaltensbeobachtungen des allgemeinen Vollzugdiensts im Jugendstrafvollzug [Diagnostic utility of structured assessment of behavioral obervations by prison warders in juvenile offender prisons]. Forensische Psychiatr Psychol Kriminol. (2017) 11:163–74. 10.1007/s11757-017-0416-5

[29] Shedler J Westen D. Refining the measurement of Axis II: a Q-sort procedure for assessing personality pathology. Assessment (1998) 5:335–55. 10.1177/107319119800500403 9835657

[30] Westen D Shedler J Bradley B DeFife JA. An empirically derived taxonomy for personality diagnosis: bridging science and practice in conceptualizing personality. Am J Psychiatry (2012) 169:273–84. 10.1176/appi.ajp.2011.11020274 22193534 PMC4546840

[31] Marin-Avellan LE McGauley GA Campbell CD Fonagy P. The validity and clinical utility of structured diagnoses of antisocial personality disorder with forensic patients. J Pers Disord. (2014) 28:500–17. 10.1521/pedi_2014_28_129 24511901

[32] DeFife JA Malone JC DiLallo J Westen D Assessing adolescent personality disorders with the Shedler–Westen Assessment Procedure for Adolescents. Clin Psychol Sci Pract. (2013) 20:393–407. 10.1111/cpsp.12049

[33] Cicchetti DV Guidelines, criteria, and rules of thumb for evaluating normed and standardized assessment instruments in psychology. Psychol. Assess. (1994) 6:284.

[34] Shedler J Westen D (2004). Dimensions of Personality Pathology: An Alternative to the Five-Factor Model. Am J Psychiatry 161:1743–1754. 10.1176/ajp.161.10.1743 15465966

[35] Löffler-Stastka H Ponocny-Seliger E Fischer-Kern M Rössler-Schülein H Leithner-Dziubas K Schuster P. Validation of the SWAP-200 for diagnosing psychostructural organization in personality disorders. Psychopathology (2007) 40:35–46. 10.1159/000096388 17057423

[36] Hare RD The Psychopathy Checklist–Revised (PCL-R). Toronto, CA: MHS (2003).

[37] Andrews DA Bonta J The Psychology of Criminal Conduct (5th ed.). New Providence, NJ: Lexis Nexis (2010).

[38] Andrews DA Bonta J LSI-R: The Level of Service Inventory-Revised. Toronto, CA: Multi-Health Systems (1995).

[39] Robbé MDV Vogel VD Douglas KS Risk factors and protective factors: a two-sided dynamic approach to violence risk assessment. J Forensic Psychiatry Psychol. (2013) 24:440–57. 10.1080/14789949.2013.818162

[40] Dahle KP Harwardt F Schneider-Njepel V Andrews D Bonta J Inventar zur Einschätzung des Rückfallrisikos und des Betreuungs-und Behandlungsbedarfs von Straftätern (LSI-R), Manual [LSI-R: German version]. Göttingen: Hogrefe (2012).

[41] Webster CD Douglas KS Eaves D Hart SD HCR-20: Assessing Risk of Violence (Version 2). Vancouver, CA: Simon Fraser University (1997).

[42] Müller-Isberner R Jöckel D Cabeza SG Die Vorhersage von Gewalttaten mit dem HCR-20 [The prediction of violence with the HCR-20 scheme]. Haina, GER: Institut für Forensische Psychiatrie (1998).

[43] Dahle KP. Strengths and limitations of actuarial prediction of criminal reoffence in a German prison sample: a comparative study of LSI-R, HCR-20 and PCL-R. Int J Law Psychiatry (2006) 29:431–42. 10.1016/j.ijlp.2006.03.001 16780950

[44] MacCallum RC Widaman KF Zhang S Hong S Sample size in factor analysis. Psychol Methods (1999) 4:84–99. 10.1207/S15327906MBR3604_06 26822184

[45] O'connor BP. SPSS and SAS programs for determining the number of components using parallel analysis and Velicer's MAP test. Behav Res Methods Instrum Comput. (2000) 32:396–402. 10.3758/BF03200807 11029811

[46] Hayton JC Allen DG Scarpello V Factor retention decisions in exploratory factor analysis: a tutorial on parallel analysis. Organ Res Methods (2004) 7:191–205. 10.1177/1094428104263675

[47] Fabrigar LR Wegener DT Exploratory Factor Analysis. Oxford: University Press (2011).

[48] Tabachnick BG Fidell LS Using Multivariate Statistics (6th ed.). Boston, MA: Pearson (2013).

[49] Rice ME Harris GT. Comparing effect sizes in follow-up studies: ROC Area, Cohen's d, and r. Law Hum Behav. (2005) 29:615–20. 10.1007/s10979-005-6832-7 16254746

[50] Nunnally J Psychometric Methods (2nd ed.). New York, NY: McGraw Hill (1978).

[51] Fleiss JL Levin B Paik MC Statistical Methods for Rates and Proportions (3rd ed.). Hoboken, NJ: Wiley and Sons (2003).

[52] Tzelgov J Henik A Suppression situations in psychological research: Definitions, implications, and applications. Psychological Bulletin (1991) 109:524–536. 10.1037/0033-2909.109.3.524

[53] Krueger RF McGue M Iacono WG The higher-order structure of common DSM mental disorders: internalization, externalization, and their connections to personality. Pers Individ Dif (2001) 30:1245–59. 10.1016/S0191-8869(00)00106-9

[54] Krueger RF Markon KE. Reinterpreting comorbidity: a model-based approach to understanding and classifying psychopathology. Annu Rev Clin Psychol. (2006) 2:111–33. 10.1146/annurev.clinpsy.2.022305.095213 17716066 PMC2242354

[55] Kowalski RM Behaving Badly: Aversive Behaviors in Interpersonal Relationships. Washington, DC: APA (2001).

[56] Paulhus DL Williams KM The dark triad of personality: Narcissism, Machiavellianism, and psychopathy. J Res Pers. (2002) 36:556–63. 10.1016/S0092-6566(02)00505-6

[57] Jones DN Paulhus DL The role of impulsivity in the Dark Triad of personality. Pers Individ Dif. (2011) 51:679–82. 10.1016/j.paid.2011.04.011

[58] Furnham A Richards SC Paulhus DL The Dark Triad of personality: a 10 year review. Soc Personal Psychol Compass (2013) 7:199–216. 10.1111/spc3.12018

[59] Walters GD. Predicting institutional adjustment and recidivism with the psychopathy checklist factor scores: a meta-analysis. Law Hum. Behav. (2003) 27:541–58. Available online at: https://link.springer.com/article/10.1023/A:1025490207678 14593797 10.1023/a:1025490207678

[60] Hanson RK Morton-Bourgon KE. The characteristics of persistent sexual offenders: a meta-analysis of recidivism studies. J Consult Clin Psychol. (2005) 73:1154–63. 10.1037/0022-006X.73.6.1154 16392988

[61] Zamble E Porporino FJ Coping, Behavior, and Adaptation in Prison Inmates. New York, NY: Springer (2013).

[62] Scoones CD Willis GM Grace RC. Beyond static and dynamic risk factors: the incremental validity of release planning for predicting sex offender recidivism. J Interpers Violence (2012) 27:222–38. 10.1177/0886260511416472 21859756

[63] Suhling S Rehder U Zum Zusammenhang zwischen Vollzugslockerungen, Unterbringung im offenen Vollzug und Legalbewährung bei Sexualstraftätern [On the relationship between temporary absence status, minimum security confinement, and recidivism in sexual offenders]. Forensische Psychiatr Psychol Kriminol. (2009) 3:37–46. 10.1007/s11757-008-0107-3

[64] Lehmann RJB Dahle K-P Schmidt AF Primer on the contribution of crime scene behavior to the forensic assessment of sexual offenders. Eur Psychol. (2018) 23:154–66. 10.1027/1016-9040/a000324

[65] McDougall C Pearson D Bowles R Cornick J Institutional offence behaviour monitoring as an aid to community supervision of high risk offenders: experience from multi-agency public protection arrangements. In Daffern M Jones L Shine J, editors, Offence Paralleling Behavior: A Case Formulation Approach to Offender Assessment and Intervention, Oxford: Wiley-Blackwell (2010). p. 185–202.

